# Assessment of risk factors associated with falls among the elderly in a municipality in the state of Paraíba, Brazil. A cross-sectional study

**DOI:** 10.1590/1516-3180.2018.0198120619

**Published:** 2020-01-13

**Authors:** Alba Rejane Gomes de Moura Rodrigues, José Cesar Assef, Carlos Bezerra de Lima

**Affiliations:** I BSN. Nurse and Doctoral Student of Surgical Research, Faculdade de Ciências Médicas da Santa Casa de São Paulo (FCMSCSP), São Paulo (SP); and Instructor, Unidade Acadêmica de Enfermagem (UAENF), Universidade Federal de Campina Grande (UFCG), Cajazeiras (PB), Brazil.; II MD. Associate Professor, Department of Surgery, and Director of Emergency Services, Faculdade de Ciências Médicas da Santa Casa de São Paulo (FCMSCSP), São Paulo (SP), Brazil.; III BSN, MD. Nurse, Nursing Department, Universidade Federal do Rio de Janeiro (UFRJ), Rio de Janeiro (RJ); Teacher of Postgraduate Program, Faculdade Brasileira de Ensino Pesquisa e Extensão (FABEX), João Pessoa (PB), Brazil.

**Keywords:** Risk factors, Aged, Accidental falls

## Abstract

**BACKGROUND:**

Falls among the elderly are one of the main public health problems that have direct consequences for their health. They reduce these individuals’ autonomy and functional independence.

**OBJECTIVE:**

The objective of this study was to evaluate the risk factors associated with falls among elderly people enrolled within primary healthcare.

**DESIGN AND SETTING:**

Cross-sectional study conducted at primary healthcare units in the municipality of Patos, state of Paraíba, Brazil.

**METHODS:**

The Fall Risk Score and Timed Up and Go (TUG) test were used for evaluating the risk of falling among 316 elderly individuals. The independent variables used were sociodemographic and health conditions, while the dependent variable was the frequency of falls on the same level, over the course of previous years. The descriptive statistical tests used were the chi-square and Mann-Whitney tests.

**RESULTS:**

Occurrence of falls was reported by 211 of the 316 participants, representing a prevalence of 66.8% with confidence interval 61.6-72.0. The logistic regression results showed, after adjusting for all variables included in the model, that only the variables of vestibular disorders, self-assessed health status and dizziness/vertigo (trend) were significant (P ≤ 0.05). Most of the elderly participants had two or more associated pathological conditions. The participants were predominantly female (68.4%).

**CONCLUSIONS:**

Higher occurrence of falls was observed among female elderly individuals who suffered recurrent falls, had had low levels of schooling, presented comorbidities, had comorbidities and made use of drugs. These conditions predisposed these individuals to greater vulnerability to the risk of falls.

## INTRODUCTION

The numbers of elderly people are growing rapidly. Both the community collectively and healthcare professionals, family members and caregivers individually need to reflect on their commitment towards dealing with the changes that occur as people become older. Aging is a natural, progressive and irreversible process that directly interferes with biological and functional conditions. Hence, one-off actions aimed at promoting healing and/or rehabilitation are insufficient.^1^

Statistical data show that Brazil has 20.6 million elderly people, representing 10.8% of its total population. By 2060, this country’s elderly population is expected to reach 58.4 million (26.7% of the total population).^2^ Accordingly, guidance for actions to diminish the risk factors for falls in home settings will become very relevant.

The incidence of falls varies across countries. Studies conducted in Latin America and the Caribbean region have identified that, on an annual basis, the proportion of older adults suffering falls ranges from 21.6% in Barbados to 34% in Chile.^3^ These data emphasize that there is a need to take a more accurate look into the settings within which elderly people live. This is a major challenge, with regard both to identifying the elderly people who are at risk and to planning preventive strategies, irrespective of geographical location.

Falls are the sixth leading cause of death among people over 65 years old. They make these individuals fragile, insecure and unable to perform their daily activities for fear of falling again.^4^ Both intrinsic factors (physiological changes stemming from aging itself; presence of morbidities; and deficits in balance, vision, hearing or gait) and extrinsic factors (environmental risks due to poor lighting or inadequate or slippery floors; risk-prone behaviors, such as going upstairs and downstairs; and routine activities of daily living) play important roles. The multifactorial circumstances of this interaction of factors may predispose this group of vulnerable individuals to falls.^5^^,^^6^

Occurrences of falling have recently become a public health problem due to the complications resulting from them. They place a burden on the healthcare system and bring about physical mobility limitations. According to the Brazilian Ministry of Health, about 30% of people aged over 65 fall at least once a year.^7^ In a study conducted in Australia among elderly people living in the community, it was reported that 2-6% of falls were associated with fractures and approximately 1% of falls were associated with hip fractures.^8^

The municipality of Patos, in the state of Paraíba, was the scenario for the present study. A total of 426 hospital admissions due to falls were registered between January 2008 and December 2015, which accounted for 6.9% of all hospitalizations.^9^ In addition to this high percentage of hospital admissions due to falls, this municipality does not have resources for rehabilitation, and no study on falls among the elderly had previously been conducted in this municipality. Recognizing the most vulnerable groups and acting so as to prevent falls by engaging the efforts of an interdisciplinary team can contribute towards minimizing these events.^9^

## OBJECTIVE

The objective of this study was to assess the risk factors associated with falls among elderly people enrolled within primary healthcare in the municipality of Patos, Paraíba, Brazil.

## METHODS

This was a quantitative cross-sectional study carried out between April and December 2016 at 40 family health units (Unidades de Saúde da Família, USF), among which 38 were located in the urban area and two in the rural area of the municipality of Patos, in the state of Paraíba, Brazil. The population sample size was calculated by considering a prevalence of falls of 30%. This was in accordance with the national prevalence of falls in Brazil^1^^,^^8^ for a finite population of 13,453 elderly people. It was thus determined that a sample of 316 individuals would be needed.

*A posteriori* power calculations indicated that all the variables that were shown to be significantly associated in the logistic regression model had a statistical power of at least 94.8% for the sample size used in our study and the effect size we found (odds ratio-based).

The elderly participants were selected across the healthcare districts of the municipality by means of systematic sampling and by organizing a single listing of these districts. There are four healthcare districts in the municipality of Patos, each with ten healthcare units. The average number of elderly people per district was 3,200. The proportion of the sample across each of the four districts was calculated on this basis.

The sample selection process was then performed according to areas and micro-areas where community health agents acted, which, in turn, belonged to various census tracts (districts). Each community health agent had a spreadsheet containing data on their corresponding microarea, regarding street names, numbers of households per street with their respective full address and the estimated number of elderly people per household.

A draw was conducted among each five elderly people who had been selected to participate, based on the spreadsheet data. Thus, it was possible for more than one elderly person in the same household to participate in this study, given that individuals were selected, rather than their household. There were occurrences of situations in which two or more elderly people from the same household were selected to participate in the study, but only one of them was actually interviewed. Also, there were cases in which all the elderly people selected from a single household were interviewed. Overall, an average of 8-10 elderly people were selected from each healthcare unit. We did not have any dropouts or refusals.

The inclusion criteria were that the participants needed to be ≥ 60 years of age and be resident in an area assigned to the units in question. Elderly people presenting some cognitive deficit, according to information provided by the community health agents, and those making use of any kind of device to aid walking, or a wheelchair, were excluded.

For data collection, two types of instruments were used. Firstly, a questionnaire was applied to gather sociodemographic data, with questions on gender, age, marital status, level of schooling, income and current occupation, among other data; and to gather self-reported information on the elderly participants’ morbidities. Secondly, two fall risk assessment instruments were used: the Fall Risk Score, adapted from a study by Shiaveto,^10^ which uses five criteria to evaluate the risk of falls among the elderly population; and the TUG (Timed Up and Go) test, which assesses gait and balance.

In the TUG test, the time spent (in seconds) for the elderly individual to stand up from a seated position in a chair, walk a 3-meter distance, turn around, walk back towards the chair and sit down again was recorded. The individuals underwent this test once beforehand so that they could become familiar with it. They were given no assistance whatsoever as they took the test.

The Fall Risk Score evaluates the following: 1- Whether the elderly individuals had had any falls previously; 2- Whether they were using any medication; 3- Whether they presented any sensory deficits; 4- Their mental state; and 5- Their gait.

The following independent variables were selected for this study: sociodemographic variables: (age, gender, marital status and schooling level); and health status variables: self-perception of health status, living alone, walking without difficulty, presence of changes (comorbidities), types of changes (comorbidities), medications (quantity and type) and other health hazards (dizziness/vertigo). The dependent variable was the frequency of falls from the same level over recent years.

The participants’ cognitive ability was evaluated with the help of the Functional Activities Questionnaire (FAQ), devised by Pfeiffer.^11^ This scale was used in its version validated for use in Brazilian Portuguese. Cognitive ability was categorized as follows: absence of cognitive decline, or mild, moderate or severe critical decline. Elderly people with severe cognitive decline were excluded.

The interviews to administer the questionnaires were conducted by one author of the present study (ARGMR) and by three interviewers from the nursing undergraduate course who were enrolled in a course module on elderly people’s healthcare and were trained and instructed on how to administer the questionnaire and its assessment grading scales. The interviews were conducted by means of visits to the elderly people’s homes, which had previously been scheduled by the health agent in charge of the area where these elderly people lived.

Following data collection, a database was prepared in an Excel spreadsheet. To carry out the analysis, the database was exported from the Excel spreadsheet to the Statistical Package for the Social Sciences (SPSS) software, version 17.0. The data were then subjected to descriptive analysis (absolute and percentage frequencies), and the normality of age distribution was checked by means of the Shapiro-Wilk test, which showed that this variable did not follow normal distribution (P < 0.05).

Subsequently, in order to check the association between occurrences of falls and each of the categorical variables, chi-square tests or Fisher’s exact tests, as appropriate, were performed. To check whether there was an association between occurrences of falls and age, the non-parametric Mann-Whitney test was used. The variables that were found to be significantly associated with occurrences of falls were then fed into a logistic regression model to check the independent predictors of occurrences of falls, after adjustment for the other variables of the model. The significance level adopted for the statistical analysis was P < 0.05.

This study was approved by our institution’s research ethics committee, through decision no. 962,318 (CAAE 38956414.00000-5181), on February 25, 2015, in accordance with Resolution No. 466/12 of Brazil’s National Health Council (Conselho Nacional de Saúde).^12^ All study participants were guaranteed that their participation would be voluntary, of their own free will, and were only included in the study after they had read and signed an informed consent statement. After informing illiterate elderly people about the objectives of the study, the person responsible for each individual in this situation who agreed to participate in the study was then asked to sign the voluntary consent form in lieu of this elderly individual.

## RESULTS

Altogether, 316 elderly people were evaluated. Out of the total number of elderly people interviewed, 68.4% were females. The most prevalent age group was from 60 to 69 years old (40.5%). The minimum age was 60 years, and the maximum age was 99 years, with a mean of 73, median of 72 and standard deviation of 9. The majority of the elderly people (64.7%) had completed primary education, while 19.9% were illiterate. Regarding their marital status, 47.5% were married, 22.8% widowed and 15.8% were divorced.

Occurrence of falls in the period between 2015 and 2016 was reported by 211 of these 316 participants, thus representing a prevalence of 66.77%, with a confidence interval (CI) of 61.6-72.0. Table 1 shows the distribution of the elderly people studied according to their sociodemographic variables and histories of falls. A significant association was found between occurrences of falls and schooling level (P = 0.042). Among the individuals who sustained falls, there was a greater proportion who were illiterate or who had only completed primary education. No significant differences were found in relation to the other demographic variables.

**Table 1.** Distribution of the elderly people studied, according to sociodemographic variables and history of falls. Patos (PB), 2016

Sociodemographic variables	Categories	No falls	With falls	P
n		n = 105	n = 211	
Gender	Male	35 (33.3)	65 (30.8)	0.744
	Female	70 (66.7)	146 (69.2)	
Marital status	Single	10 (9.5)	34 (16.1)	0.335
	Married	55 (52.4)	95 (45.0)	
	Divorced	18 (17.1)	32 (15.2)	
	Widowed	22 (21.0)	50 (23.7)	
Schooling level	Illiterate	13 (12.4)	50 (23.7)	0.042
	Incomplete primary education	55 (52.4)	101 (47.9)	
	Completed primary education	12 (11.4)	32 (15.2)	
	Incomplete secondary education	2 (1.9)	4 (1.9)	
	Completed secondary education	13 (12.4)	11 (5.2)	
	Incomplete higher education	0 (0.0)	1 (0.5)	
	Completed higher education	10 (9.5)	12 (5.7)	

With regard to the possible risk factors for occurrences of falls, comorbidities and self-assessed health status were evaluated (Table 2). Among the self-reported pre-existing chronic comorbidities or diseases, the proportions of these that showed associations with other pathological conditions were as follows: systemic arterial hypertension (81.5%), arthrosis (37.9%) and vestibular disorders (33.2%).

**Table 2.** Association between comorbidities and presence of falls. Patos (PB), 2016

Comorbidities	Categories	No falls	With falls	P
		n = 105	n = 211	
Diabetes	No	76 (72.4)	129 (61.1)	0.065
	Yes	29 (27.6)	82 (38.9)	
Hypertension	No	33 (31.4)	39 (18.5)	0.015
	Yes	72 (68.6)	172 (81.5)	
Stroke	No	104 (99.0)	201 (95.3)	0.108
	Yes	1 (1.0)	10 (4.7)	
Low blood pressure	No	100 (95.2)	206 (97.6)	0.309
	Yes	5 (4.8)	5 2.4)	
Heart disease	No	91 (86.7)	164 (77.7)	0.081
	Yes	14 (13.3)	47 (22.3)	
Neurological disease	No	103 (98.1)	201 (95.3)	0.349
	Yes	2 (1.9)	10 (4.7)	
Insomnia	No	90 (85.7)	174 (82.5)	0.567
	Yes	15 (14.3)	37 (17.5)	
Hypothyroidism	No	103 (98.1)	194 (91.9)	0.055
	Yes	2 (1.9)	17 ( 8.1)	
Depression	No	93 (88.6)	184 (87.2)	0.868
	Yes	12 (11.4)	27 (12.8)	
Arthritis	No	86 (81.9)	159 (75.4)	0.242
	Yes	19 (18.1)	52 (24.6)	
Arthrosis	No	82 (78.1)	131 (62.1)	0.006
	Yes	23 (21.9)	80 (37.9)	
Vestibular disorders	No	92 (87.6)	141 (66.8)	< 0.001
	Yes	13 (12.4)	70 (33.2)	
Incontinence	No	104 (99.0)	199 (94.3)	0.067
	Yes	1 (1.0)	12 (5.7)	
Anxiety	No	85 (81.0)	168 (79.6)	0.897
	Yes	20 (19.0)	43 (20.4)	
Osteoporosis	No	70 (66.7)	113 (53.6)	0.035
	Yes	35 (33.3)	98 (46.4)	

Self-assessed health status showed a statistically significant association with occurrences of falls among the elderly people who participated in our study. When asked about how they assessed their own health status, 45.6% considered it good; 26.6%, normal; and 26.3%, very good. A significant association was found between occurrences of falls and self-assessed health status (P < 0.001). Among the individuals who suffered falls, there were higher proportions with good and normal health status, whereas among individuals who reported not falling, there was a higher proportion with very good health status (Table 3).

**Table 3.** Distribution of the elderly people according to the occurrence of falls, in relation to the variable of health status assessment in the municipality of Patos (PB), 2016

Health status assessment	Overall		No falls		With falls		P^1^
	n	%	n	%	n	%	
Very good	83	26.3	53	63.9	30	36.1	< 0.001
Good	144	45.6	38	26.4	106	73.6	
Normal	84	26.6	13	15.5	71	84.5	
Poor	3	0.9	1	33.3	2	66.7	
Very poor	2	0.6	0	0.0	2	100	

^1^Chi-square test.

Because the elderly participants still had a fairly independent and autonomous lifestyle, they self-identified as independent. However, they suffered falls more often probably because they enjoyed walking around, given that they felt more self-confident and did not fear being exposed to risk factors (Table 3). In this study, the prevalence of falls was higher among the elderly people who self-assessed their health status as good.

Among the individuals evaluated, significant associations were found between occurrences of falls and occurrences of comorbidities and use of medications. Use of several medications was another trait found in our study among the elderly people who sustained falls (Table 4).

**Table 4.** Distribution of medication-related factors, in relation to occurrences of falls. Patos (PB), 2016

Medications	Categories	No falls	With falls	P
n		105	211	
Use of medications	No	17 (16.2)	8 (3.8)	< 0.001
	Yes	88 (83.8)	203 (96.2)	
Number of medications	1 to 2	74 (84.1)	130 (64.0)	0.002
	3 to 4	12 (13.6)	52 (25.6)	
	5	2 (2.3)	21 (10.3)	
Use of antihypertensives	No	33 (31.4)	39 (18.5)	0.015
	Yes	72 (68.6)	172 (81.5)	
Use of hypoglycemic agents/insulin agents	No	77 (73.3)	133 (63.0)	0.089
	Yes	28 (26.7)	78 (37.0)	
Use of diuretics	No	99 (94.3)	189 (89.6)	0.239
	Yes	6 (5.7)	22 (10.4)	
Use of analgesics	No	70 (66.7)	108 (51.2)	0.013
	Yes	35 (33.3)	103 (48.8)	
Use of sedatives	No	102 (97.1)	202 (95.7)	0.757
	Yes	3 (2.9)	9 (4.3)	
Use of antidepressants	No	105 (100.0)	207 (98.1)	0.306
	Yes	0 (0.0)	4 (1.9)	

Significant associations were found between occurrences of falls and use of medications, quantity of medications, use of antihypertensive drugs and use of analgesics. Among individuals who suffered falls, there were higher proportions of use of medications, quantity of medications and use of antihypertensives and analgesics.

### Logistic regression analysis

The variables that were significantly associated with occurrences of falls in the univariate analysis underwent logistic regression analysis, in which occurrence of falls was used as a dependent variable. The aim of this analysis was to identify which variables were independent predictors of occurrence of falls, with adjustment for all other variables included in the model. Because of the strong association between the variables of use of medications and dosages of medications, only the variable of use of medications was included in the final model. This decision was necessary in order to avoid multicollinearity in the model, thus allowing for thorough implementation (Table 5).

**Table 5.** Logistic regression analysis using occurrence of falls as a dependent variable. Patos (PB), 2016

Variable	Beta	SE beta	OR	95% CI lower limit	95% CI upper limit	P
(Intercept)	-3.677	1.522	0.03	0.00	0.48	0.016
Schooling level: incomplete primary education	-0.081	0.420	0.92	0.40	2.08	0.846
Schooling level: completed primary education	0.300	0.535	1.35	0.47	3.91	0.575
Schooling level: incomplete secondary education	-0.245	1.091	0.78	0.10	8.16	0.822
Schooling level: completed secondary education	-0.427	0.606	0.65	0.20	2.14	0.481
Schooling level: incomplete higher education	14.473	1455.398	Inf	0.00	NA	0.992
Schooling level: completed higher education	-0.157	0.671	0.85	0.23	3.21	0.815
Age	0.031	0.019	1.03	0.99	1.07	0.096
Systemic arterial hypertension (yes)	-0.298	1.043	0.74	0.09	6.07	0.775
Arthrosis (yes)	0.362	0.319	1.44	0.77	2.71	0.256
Vestibular disorder (yes)	0.801	0.400	2.23	1.04	5.03	0.045
Osteoporosis (yes)	0.244	0.297	1.28	0.71	2.29	0.410
Use of medications (yes)	0.570	0.640	1.77	0.51	6.38	0.373
Use of antihypertensives (yes)	0.338	1.051	1.40	0.16	11.27	0.748
Use of analgesics (yes)	-0.054	0.318	0.95	0.51	1.77	0.866
Health status assessment: good	1.261	0.348	3.53	1.80	7.06	< 0.001
Health status assessment: normal	1.696	0.438	5.45	2.35	13.19	< 0.001
Health status assessment: poor	0.634	1.400	1.89	0.13	50.31	0.650
Health status assessment: terrible	15.668	1023.691	Inf	0.00	NA	0.988
Dizziness/vertigo (yes)	0.625	0.318	1.87	1.00	3.51	0.050

SE = standard error; OR = odds ratio; CI = confidence interval.

From the logistic regression analysis, it could be seen that, after adjustment for all variables included in the model, only the variables of vestibular disorders, self-assessed health status and dizziness/vertigo (trend) were significant. Interpretation of odds ratios (OR) showed that individuals with vestibular disorders presented a 2.23-fold greater risk of falling than those without vestibular disorders. Furthermore, in relation to individuals with very good health status, individuals with good health status had a 3.53-fold higher risk of falling; and individuals with normal health status had a 5.45-fold greater risk of falling. Lastly, individuals with dizziness/vertigo had a 1.87-fold greater risk of falling, in relation to individuals without dizziness/vertigo.

The presence of vestibular disorders, imperfect self-assessed health status and presence of dizziness/vertigo were independent predictors for occurrence of falls. The other predictors, while being statistically significant when evaluated separately through logistic regression, were not statistically relevant.

## DISCUSSION

In this study, the prevalence of the occurrence of falls in the period between 2015 and 2016 was 66.77%. We found that 91.2% of the sample was using some sort of medication, mainly for controlling systemic arterial hypertension, diabetes mellitus or pain. Use of drugs and presence of diseases are two risk factors for occurrences of falls.^10^ The prevalence of falls was higher among females and older individuals with lower schooling levels.

The prevalence of falls last year (66.77%), as found in this study, can be considered high, in comparison with other Brazilian studies, in which the reported prevalence has ranged from 28% to 37.5%.^13^^,^^14^^,^^15^ In a systematic review of the literature, Sandoval et al.^16^ found that the prevalence of falls among elderly residents living in the community ranged from 15.9% to 56.3%. A lower prevalence was found in the United States, while the highest prevalence was found in Brazil. In Europe, and more specifically in Spain and Italy, the prevalence was found to be 30.5% to 31.8%.^17^ In Africa, a study conducted in Nigeria showed a prevalence of 23%.^18^ In Asia, a study conducted in China showed a prevalence of 26.4%.^19^ Differences in prevalence between studies have not been analyzed in depth and may have been caused by different study designs and varying methodologies.

With regard to self-reported pre-existing chronic comorbidities or diseases, the proportions of these that showed associations with other pathological conditions were as follows: systemic arterial hypertension (77.2%), osteoporosis (42.1%), diabetes (35.1%), arthrosis (32.6%), vestibular disorders (26.3%), anxiety (22.5%) and urinary incontinence (92.3%). It is important to emphasize that the elderly participants could choose to report the presence of more than one comorbidity. Among those that we evaluated, systemic arterial hypertension, osteoporosis, diabetes, arthrosis, vestibular disorders, thyroid problems and urinary incontinence were statistically significant for the risk of falls.

In the present study, we found that the elderly participants had low levels of schooling, and that 49.4% had not finished elementary school. This was found to be relevant regarding occurrences of falls. Lopes,^20^ in Uberaba, in the state of Minas Gerais, Brazil, also found a high percentage of low levels of schooling among elderly people living in the community, and that 43.1% of them were illiterate. The percentage in their study was slightly lower than what we found in our study, but it was still a significant predictor of falls in that population. In a cohort study conducted on 1,415 elderly people in the city of São Paulo, Brazil, Perracini and Ramos^21^ found that most of the elderly people who had had falls were illiterate and fell recurrently. Their finding was similar to our own in this study. Marin et al.^22^ and Freitas et al.^23^ also considered that the level of schooling was an important factor that deserved to be highlighted, since it might have made it more difficult to provide care services, given that such clients would be less involved in their own care and in the risk prevention process.

Development of multiple chronic pathological conditions and comorbidities are among the consequences that accompany aging, along with intake of various medications. Chronic diseases can most often cause systemic problems that require regular use of more than one medication, and this contributes towards occurrences of falls.

The presence of comorbidities is a risk factor, and systemic arterial hypertension is an aggravating factor for the risk of falls and fractures. Hence, given the high prevalence of systemic arterial hypertension in the population of the present study, there is a need for hypertensive patients to be better monitored for the pharmacological interactions among antihypertensive drugs, by medical and healthcare professionals. The aim in doing this is both to improve their health status and to reduce the occurrence of falls.^24^

Tests to assess the decline in visual function with age were not used in the present study because most of the elderly participants did not report any complaints regarding their vision. We consider this to be a weak point in the present study.

In assessing patients using Dowton’s Fall Risk Score scale,^10^ we found that among the elderly people who had reported previous falls, such reports were more frequent among those who had been using hypertensive medications. Regarding the time that these individuals took to do the TUG test, their average time was found to be 11.4 s and the standard deviation was 3.5 s. Upon applying the TUG cutoffs, most of these elderly people, i.e. 177 (56.0%) were classified as being at medium risk of falls.

Several studies have addressed medications as a major risk factor for falls. In a study carried out in Ribeirão Preto, in the state of Sao Paulo, Brazil, Fabrício et al.^25^ observed that 70% of the elderly participants were using some type of medication before falling and that 42% of them used polypharmacy. This corroborates our findings.

The main causes of falls that were self-reported by the elderly participants were the following: dizziness (42.7%), carelessness (24.2%), slipping (14.7%), imbalance (11.8%) and alcohol consumption (3.8%). We were able to group these causes into extrinsic and intrinsic factors that led to falls. The intrinsic factors of dizziness and (to a smaller but significant extent) imbalance predominated and were more prevalent than the extrinsic factors. This corroborate the findings of Fhon,^26^ who also observed higher prevalence of intrinsic risk factors. Among the extrinsic factors, carelessness and slipping can, according to Guimarães,^27^ be triggered by the aging process. This process alters gait patterns, limits the amplitude of dorsiflexion of the ankles and reduces strength.

Despite the significant aspects of falls among the elderly and their associated factors that were identified in our study, memory bias may have interfered in the results from this evaluation, given that the occurrences of falls recorded here were based on self-reports. The cross-sectional design was also one of the limitations of the study, since this design does not allow for direct assessment of the possible cause and effect relationship between the predictors of falls. Nevertheless, the direction and magnitude of these associations may contribute towards identifying important risk factors that are useful for preventing occurrences of these falls among the elderly at home.

## CONCLUSION

Falls and fractures among elderly people are multifactorial. This study showed that the prevalence of falls was high and associated with comorbidities. High blood pressure was found to be a preponderant risk factor. Therefore, interdisciplinary actions to meet the needs of the elderly are essential for preventing falls
